# Livestock–Carnivore Coexistence: Moving beyond Preventive Killing

**DOI:** 10.3390/ani12040479

**Published:** 2022-02-15

**Authors:** Skarleth Chinchilla, Eric van den Berghe, John Polisar, Constanza Arévalo, Cristian Bonacic

**Affiliations:** 1 Department of Ecosystems and Environment, School of Veterinary Medicine, Pontifical Catholic University of Chile, Santiago P.O. Box 782044, Chile; sjchinchilla@uc.cl (S.C.); coarevalo@uc.cl (C.A.); 2 Department of Agricultural Sciences and Production, Zamorano University, Tegucigalpa P.O. Box 93, Honduras; 3 Department of Environment and Development, Zamorano Biodiversity Center, Zamorano University, Tegucigalpa P.O. Box 93, Honduras; eric.vandenberghe050@gmail.com (E.v.d.B.); polisarejr@gmail.com (J.P.)

**Keywords:** livestock predation, free grazing, Reserva del Hombre y la Biósfera del Río Plátano, Miskitus, SDG2, SDG 15

## Abstract

**Simple Summary:**

Sustainable Development Goals for 2030 indicate that zero hunger (SDG 2) and halting biodiversity loss (SDG 15) are key priorities. Livestock management practices that allow coexistence with top predators are necessary to achieve both objectives in Latin America. This article addresses the situation in local indigenous communities near key biodiversity hotspots that protect top predators in Mesoamerica. Results show that livestock predation is related to landscape variables and human influence. Jaguar and puma conservation requires measures that facilitate human–carnivore coexistence and comply with SDG 2 and SDG 15. This study serves as a baseline to provide livestock management recommendations that mitigate the prevalent conflict with jaguars and pumas to reconcile SDG 2 with SDG 15.

**Abstract:**

Livestock predation is a global problem and constitutes the main source of conflict between large carnivores and human interests. In Latin America, both jaguar and puma are known to prey on livestock, yet studies in Mesoamerica have been scattered and few have been carried out in Honduras. We interviewed ranchers in a biosphere reserve where jaguars and pumas are present. Local indigenous communities reported livestock predation (average annual loss of 7% from 2010–2019), with preventive and retaliatory killing as their main actions against predation by the jaguar and puma. Other sources of cattle loss included diseases and theft. The extensive management system (free grazing) lets cattle access forests where predators are more common. We found that livestock predation is not random, but rather, related to landscape variables and human influence. Sites farther from human influence and closer to forest cover were more susceptible to predation. Jaguar and puma persistence in the biosphere reserve will require measures that facilitate human–carnivore coexistence and comply with Sustainable Development Goals (SDG) 2 and 15 (zero hunger and biodiversity conservation). We propose management practices to mitigate livestock predation in the presence of large carnivores based on examples of proven human–carnivore coexistence in Venezuela, Brazil, Paraguay, and Nicaragua, such as improving the spatial arrangement of livestock (maintaining a distance from forest areas) and the incorporation of confinement pens for young calves (at least the first three months of life) and their mothers. If the pens are built close to the property’s house and have constant surveillance and/or dogs, the results are likely to be more effective. Deploying these proven tools may help change the current negative perception of ranchers towards large carnivores that is essential to conservation under the aims of SDG 15. We recommend government policies and support aimed to strengthen livestock health to increase productivity and to reduce their vulnerability to predation. Finally, this study represents a baseline to understand the magnitude of the human–carnivore conflict over cattle in one of the largest biosphere reserves in Mesoamerica.

## 1. Introduction

In 2015, 17 Sustainable Development Goals (SDGs) were created as part of the 2030 Agenda for Sustainable Development, with the objective of ending poverty while tackling climate change and protecting the natural world [1]. SDG 15 focuses on protecting life on land, specifically, stopping biodiversity loss and protecting, restoring, and promoting the sustainable use of terrestrial ecosystems. This includes eliminating threats to terrestrial ecosystems and biodiversity, such as human–carnivore conflict, which is considered a primary driver of large carnivore population declines worldwide, including within protected areas [2]. Furthermore, as keystone predators, large carnivores play an important role in landscape conservation and ecosystem regulation [3], making them essential to the conservation of terrestrial ecosystems.

Livestock predation is a global problem and constitutes a source of conflict between large carnivores and human interests [4,5]. SDG 2 aims to achieve food security and end hunger throughout the world [1]. Throughout Latin America and the Caribbean, farming has significant potential to contribute to food security. Consequently, finding solutions to livestock predation is an important step in increasing food security in the region, in addition to stemming biodiversity loss. The severity of the human–carnivore conflict tends to increase with predator body mass [6], and the jaguar (*Panthera onca*) and puma (*Puma concolor*), the largest terrestrial predators in Latin America, are the central actors in human–carnivore conflicts within the region. Both species have been reported to prey on livestock throughout Latin America [7,8,9,10]. Predation rates tend to be higher in places where there is a permanent or seasonal depletion of natural prey [11] and livestock predation has often been found to be higher in areas with nearby forest cover [7,12]. Thus, human settlements along the edges of protected areas increase interactions between large carnivores and human activities [13] and tend to increase the risk of predation if livestock move freely into the forest [2].

The IUCN red list classifies the jaguar as ‘near threatened’ and the puma as being of ‘least concern’ throughout their entire range [14,15]. However, the conservation status of both species varies regionally and is threatened by their persecution and elimination in retaliation for livestock predation [12], as well as their preventive killing to avoid any potential livestock predation. Argentina, Brazil, Colombia, Ecuador, Honduras, Mexico, Panama, and Paraguay have all developed National Action Plans to perform national assessments of the threats to jaguars and their conservation challenges [16]. Nonetheless, there is a lack of consistency regarding their effective implementation, as is the case with Honduras, whose plan was developed in 2011, but due to lack of funding, the country’s plan has only been partially implemented and a coordination committee has yet to be established [16]. While no specific policies exist in Honduras to protect the puma, the species benefits to a certain extent from policies meant to protect jaguars. Lethal control methods (persecution and elimination) are widely practiced despite being illegal throughout most of the species’ range (including Honduras). Pumas, being less threatened, are less rigorously protected. However, the implementation of appropriate management practices in livestock production has been shown to reduce predation rates [17,18,19,20,21]. The use of electric fences, guard dogs, water buffalo, and control of calf birth periods are some of the conflict mitigation strategies that have been implemented across the region [19,21].

Livestock predation by jaguars and pumas has been extensively characterized in South American countries, particularly in Brazil [8,12,22], Argentina [23,24,25], Chile [26,27], Venezuela [11], Colombia [28], and Bolivia [10], but very few research studies have been carried out in Mesoamerica. Despite lacking extensive research regarding conflicts between large carnivores and livestock, the region harbors both predators and diverse indigenous communities. It is estimated that within Mesoamerica, Honduras may have the third largest jaguar population following Mexico and Nicaragua, holding about 12% of the Mesoamerican jaguar population [16]. Honduras also shares the second largest Jaguar Conservation Unit (JCU) in Mesoamerica with Nicaragua [29].

In Honduras, pilot studies have reported livestock predation by large carnivores in human settlements located in the cultural and buffer zones of “La Reserva del Hombre y la Biósfera del Río Plátano” (RHBRP) [30,31,32]. However, the specific characteristics of human–carnivore conflict have minimally been studied in Honduras. Consequently, the country can benefit from exploratory research to provide baseline information on the magnitude of the conflict, as well as from strategies to reduce the predation of livestock and ensure the conservation of the ecosystems inhabited by jaguars and pumas. Therefore, we aimed to understand the key features of the conflict between predators and livestock management and its consequences. We studied how livestock production is managed by several local communities near a biosphere reserve that harbors pumas and jaguars. We estimated livestock predation during the last decade, along with other causes of livestock losses, measured as annual losses associated with predation by jaguars and pumas. Finally, we describe the attitudes of local ranchers towards predators and suggest actions to foster coexistence between predators and extensive livestock production. The final aim of this study is to contribute to the reconciliation of Sustainable Development Goals 2 and 15 in areas with local indigenous communities in Honduras as a working example for other communities in forested biomes in Latin America.

## 2. Material and Methods

### 2.1. Study Area

The study was carried out in the department of Gracias a Dios (16,997 km²), also known as the Muskitia Hondureña. It is located in the life zone of the very humid subtropical forest with altitudinal ranges between 10–800 m [33]. Annual precipitation is 2200–3000 mm, with a dry season between December and April and rain from May to November, with average annual temperature ranging between 22 and 27 °C [34]. It is divided into three major subregions: the Atlantic rainforest, the pine savannas, and the mangrove forests [35]. In addition, it has protected areas of great value for biodiversity conservation and for the survival of indigenous people (Miskitus, Tawahkas, and Pesh) and Afro-Honduran peoples (Garífunas). The Muskitia Hondureña is part of the second largest Jaguar Conservation Unit (JCU) in Mesoamerica [29] and has jaguar populations that are seriously threatened [36].

**Figure 1:**
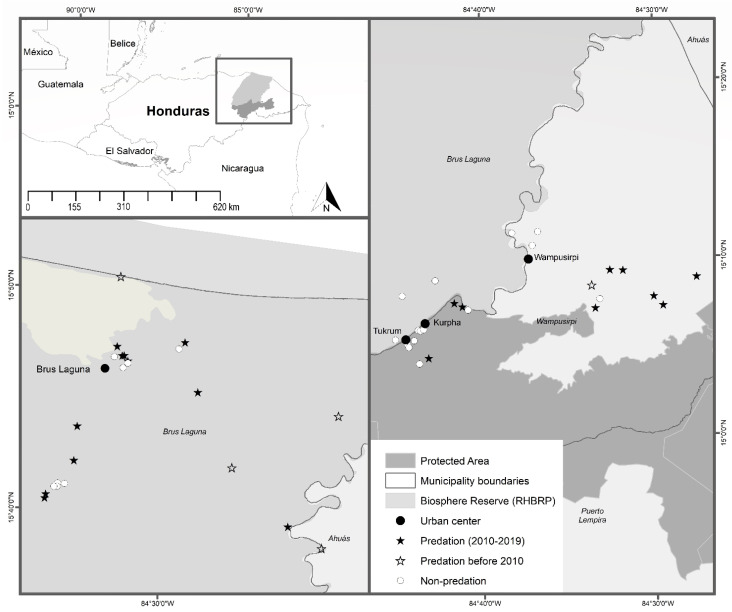
The study area showing the ranches visited in Brus Laguna and Wampusirpi communities. Black stars correspond to the location of the surveyed ranches that reported predation events between 2010–2019. Open stars correspond to the location of the surveyed ranches that reported predation events before 2010 and the white dots correspond to the location of the surveyed ranches that did not report predation events. Urban centers of communities are represented by black dots.

This study was conducted in two municipalities in “Gracias a Dios” (Figure 1), which are title lands under Territorial Councils and recognized as indigenous Miskitu territory [37]: (i) “Brus Laguna” (3291 km^2^), which is encompassed by the RHBRP, and is part of the DIUNAT Territorial Council; (ii) “Wampusirpi” (2519 km^2^), partially overlapping with the RHBRP and is part of the BAKINASTA Territorial Council. The RHBRP is one of the most important protected areas in the Mesoamerican Biological Corridor and the largest in Honduras, encompassing more than 8300 km^2^. The RHBRP was declared a UNESCO World Heritage Site and Biosphere Reserve in 1982.

### 2.2. Data Collection

We used a case study approach, a method that is widely used to study conservation conflicts [38]. During 2020, we conducted 50 interviews with farmers following their verbal consent. Respondents were anonymous to protect their identity and confidentiality, and all interviews were only used for the purpose of this study. The interviews were designed to provide information about respondent and property profile, livestock type and size, challenges of livestock production, livestock predation by jaguars and pumas from 2010–2019, rancher response to predation, and perceptions of lethal and non-lethal methods to mitigate livestock predation by large carnivores. In the BASKINASTA Territorial Council, we used an existing database of local ranchers to find participants for the study. Given the lack of such a database in the DIUNAT Territorial Council, we adopted the snowball sampling method [9,38], in which selected ranchers recruited other ranchers to participate in the study. This method is widely used in qualitative research, given that it facilitates reaching vulnerable and difficult-to-access groups. Surveys were conducted as semi-structured interviews with informal in-person dialogue with livestock owners and workers. Respondents who reported livestock predation events were shown images of carnivores present in the area. Consequently, ranchers visually identified carnivores they considered to be responsible for the predation of their livestock. The reliability of reports was evaluated based on the evidence described by respondents, such as carnivore tracks left near the predation site, signs of a struggle, bite marks on livestock throats or necks, claw marks, drag marks, and partially consumed bodies covered with leaves or other materials to hide them from other predators [7,21].

The interviews consisted of 14 questions, or 30 if the respondent acknowledged the occurrence of livestock predation. We gathered information on respondent profile (respondent’s role on the property, length of residency on the property, age, gender, education level, and indigenous group with which they associated), property profile (property location, size, and use), and challenges for livestock production (type of livestock on property, estimates of livestock units per year and annual losses, main challenges for livestock production, whether respondent had experienced livestock predation, and type of livestock management implemented). If respondents expressed having experienced livestock predation on their property, we also gathered information on such events, asking respondents: (i) whether they considered predation to be their main cause of livestock loss, (ii) to provide estimates of annual livestock losses due to predation and the responsible carnivores, (iii) whether they considered predation events to have increased or decreased over time, (iv) evidence they used to determine livestock predation by large carnivores, (v) evidence they used to determine if livestock died of natural causes before being consumed by large carnivores or if predation was the main cause of death, (vi) whether respondent could differentiate between puma and jaguar attacks, (vii) what factors they considered in differentiating between puma and jaguar attacks, (viii) time of year most predation events were observed, (ix) time of day most predation events were observed, and (x) locations on property where most livestock predation events occurred. Finally, respondents were asked a series of questions to gauge their perceptions of large carnivores and their use of lethal and non-lethal methodologies to mitigate livestock predation (see Supplementary Materials for all questions used in interviews). 

### 2.3. Landscape Features

Properties were described in terms of landscape characteristics, for which the geographic coordinates (X, Y) of the paddocks where animals graze were obtained [10,39]. Using official land cover maps created by the National Institute of Forest Conservation and Development, Protected Areas and Wildlife (Instituto Nacional de Conservación y Desarrollo Forestal, Áreas Protegidas y Vida Silvestre, ICF, Tegucigalpa, Honduras), we calculated the percentage of forest cover within a 3.6 km buffer from the center of the paddock for each study site (40 km^2^) [10]. This area was selected based on reports of the minimum home range of jaguars in similar ecosystems [40]. We also calculated the Euclidean distance from the center of the paddock to the forest edge [7,39] and from the center of the paddock to the center of the nearest community [8]. All spatial calculations were conducted using ArcGIS version 10.5.

### 2.4. Statistical Analysis

Of the 50 interviews conducted, six interviews were discarded from the study due to lack of access to some of these properties and respondents reporting predation of livestock by large carnivores before 2010. The information obtained from the remaining 44 interviews was coded into numerical categories for statistical analysis. We carried out a descriptive analysis of respondent and property profiles. To calculate annual livestock losses due to predation, respondents reported the size of livestock herds (cattle, horses, pigs, and sheep) on their properties and the minimum and maximum number of livestock predated per year [31] from 2010–2019. We used the minimum and maximum values to calculate the average annual loss due to predation in the study area. In addition, respondents reported the time of day and time of year when livestock predation was most frequent. The time was classified as day (06:00–17:59) and night (18:00–05:59), and the time of year as either dry season (December–April) or rainy season (May–November) [34]. We used the chi-square goodness of fit test (*p* < 0.05) to determine if the time of day, time of year [7], and type of livestock predated were the same across categories.

We used Fisher’s exact test (*p* < 0.05) to assess whether the opinion of how to reduce economic losses from predation was associated with the profile of the respondent and herd size [8].

To determine livestock predation (response variable) in relation to possible explanatory variables, we used a logistic regression model (GLM) for a binomial distribution (Table 1) [7,8]. We created a binary predation variable (1 = presence of predation and 0 = absence) for each respondent (*n* = 44). The explanatory variables used were percentage of forest cover, Euclidean distance to forest cover, Euclidean distance to the center of the community, property size, and herd size. To avoid collinearity between the explanatory variables, we calculated a Spearman correlation coefficient (r < 0.50) [41] before adding variables to the models. Property size was excluded because it showed a positive correlation with Euclidean distance to the center of the community (r = 0.58; *p* = 0) and with herd size (r = 0.52; *p* = 0).

We tested the overall model fit for a subset of regression parameters using maximum likelihood estimation and Akaike’s information criterion (AIC). We developed a set of candidate models a priori, including a global model with all parameters (K = 5) and with the link function ‘logit.’ We used the second order ‘corrected’ AIC (AICc) for small samples, the top delta AICc (DAICc proximity to zero), and greatest AICc weights (w) to select the best-fit model [7,8]. Lastly, we used a Hosmer–Lemeshow goodness of fit test to examine the selected model (*p* < 0.05) [7].

We used the ‘stats’ package for Spearman and GLM correlation analyses. Fisher’s exact test and chi-square test were implemented using the ‘mass’ package. Furthermore, we used the ‘AICcmodavg’ package to classify AIC-based models and the ‘generalhoslem’ package to examine the goodness of fit of the selected model. All statistical analyses were performed in RStudio (2020).

## 3. Results

### 3.1. Property Profile

The 44 properties considered in this study cover a total area of 10,944 ha. Average property size was 249 ha (SD = 349), the minimum was 4 ha, and the maximum 1410 ha. Property use included livestock (39%), crop production (6%), and forest cover (protection) (55%). The average herd size was 45 livestock units (SD = 32), with a minimum of 6 and a maximum of 153 livestock. In total, 100% of the properties (*n* = 44) had cattle (dual purpose dairy and beef cattle), 77% (*n* = 34) had horses, 34% (*n* = 15) had pigs, and 27% (*n* = 12) had sheep.

### 3.2. Respondent Profile

Most of the interviews were conducted with property owners (91% of respondents, *n* = 40) and only 9% (*n* = 4) were property employees. Of all the respondents, 20% (*n* = 9) reported a residence time greater than 20 years on their properties, 46% (*n* = 20) reported 10–20 years, 20% (*n* =9) reported 5–10 years, and 14% (*n* = 6) less than 5 years. Forty of the respondents were male and four were female. The average age of the respondents was 49 years (SD = 15), with a minimum of 23 and a maximum of 84 years. A total of 91% of the respondents classified themselves as belonging to the Miskitu indigenous people and 9% as mixed background. Education levels were varied (30% completed primary school, 27% secondary school, 32% higher studies, and 11% no formal education).

### 3.3. Challenges of Livestock Production

**Figure 2:**
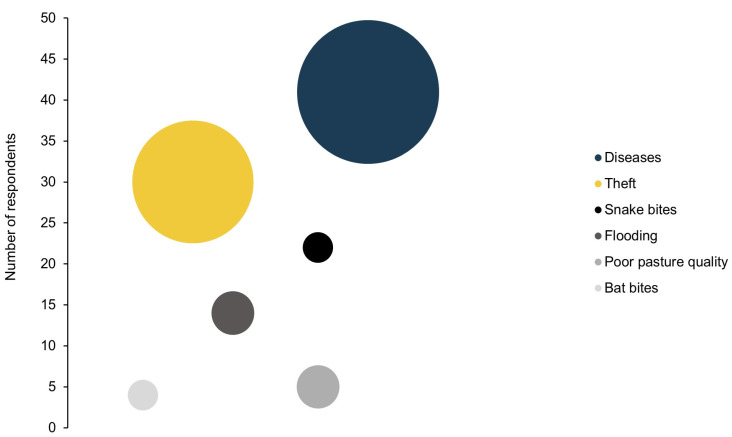
Main livestock challenges reported by ranchers. Bubble sizes are proportional to the number of respondents ranking a factor as the most important challenge, while the value on the vertical axis corresponds to the number of respondents listing that factor among the challenges of their livestock production.

Livestock extensive management is a challenging task in seminatural and remote rural areas. A main problem is herd health as 93% of the respondents (*n* = 41) mentioned diseases as a main cause of livestock losses. Respondents recognized that sanitary management is deficient due to limited access to supplies and poor veterinary knowledge. Livestock theft was mentioned by 70% of the respondents (*n* = 31) and 36% (*n* = 16) reported it as the main challenge to their livestock production. Snake and bat bites, livestock predation, floods, droughts, poor pasture quality, and pre- and postpartum problems were reported as well. However, no respondent reported drought, pre- and postpartum problems, or predation as their most significant challenge (Figure 2).

### 3.4. Livestock Predation by Large Carnivores

Current cattle management practices include free grazing near forests and riverbanks, with little supervision from herders, and there is a lack of corrals and night enclosures. Nearly half of the respondents (48%, *n* = 21) reported livestock predation by large carnivores from 2010–2019. In addition, an average annual loss of 7% of livestock from 2010–2019 was attributed to predation by jaguars and pumas. Likewise, it was reported that the type of livestock with the most predation events was cattle (χ^2^ = 73.31, *p* < 0.001), constituting 59% of preyed livestock (*n* = 68), followed by sheep (*n* = 24, 21%), pigs (*n* = 13, 11%), and horses (*n* = 11, 9%). Jaguar and puma livestock predation was grouped because only 29% of the respondents (*n* = 6) claimed to recognize the difference between the two types of attack. The reliability of each incident was assessed based on the evidence used by respondents to identify predation by large carnivores. Partially consumed bodies, carnivore tracks near the predation event, claw marks, drag marks, and neck and throat bites were the most frequently mentioned (Table 2).

A large number of respondents (81%, *n* = 17) declared that predation by jaguars and pumas occurred from 6 p.m. to dawn (χ^2^ = 10.71, *p* < 0.001). Additionally, 56% (*n* = 12) stated that predation by large carnivores occurred year-round, reporting 44% of attacks during the dry season (*n* = 9) and the remainder during the rainy season (χ^2^ = 0.43, *p* = 0.51). According to the respondents, predation frequently occurred along forest edges, riverbanks, and in grazing paddocks (Table 2).

Livestock far from human settlements and near forest edges experienced greater predation (K = 3, AICc = 55.97, *w* = 0.29) (Table 3), with no evidence of poor fit in the model (Hosmer–Lemeshow test; *p* = 0.09). On the other hand, livestock herd size was determined to be a weak predictor of the probability of livestock predation.

### 3.5. Perceptions and Measures to Mitigate Livestock Predation

The respondents reported the use of lethal and non-lethal methodologies to mitigate livestock predation. The practices mentioned were the use of guard dogs, herd vigilance, active chase and retaliatory killing of carnivores, and grouping livestock close to human facilities. Most ranchers (95%) who expressed having experienced livestock losses due to predation, declared that the implementation of these practices reduced the predation of livestock by large carnivores.

Regarding opinions on reducing economic losses due to livestock predation, 43% of respondents expressed their belief that eliminating large carnivores was the most effective way to reduce economic losses, 33% mentioned financial compensation for livestock losses, and 24% recognized that management practices on their properties required improvement to prevent livestock predation from occurring in the first place. Opinions on how to reduce economic losses from livestock predation were independent of respondent age (*p*-value = 0.97), education level (*p*-value = 0.54), and livestock units on the property (*p*-value = 0.77).

A total of 57% of respondents reported knowing someone who was responsible for the killing of at least one jaguar and/or puma and 24% voluntarily reported having eliminated at least one of these large carnivores, with the objective of preventing future livestock predations.

## 4. Discussion

Livestock predation by large carnivores results in important economic losses, especially in poor communities living in remote areas near wilderness, where livestock operations are small yet constitute a primary economic activity [42]. In recent years, rapid uncontrolled expansion of the agricultural and livestock frontier has been reported in the Muskitia Hondureña, resulting in deforestation of protected areas in the biosphere reserve [29]. Our results indicate that livestock predation poses a challenge to livestock production in the study area, though not the most significant challenge, and that landscape variables, human influence, and husbandry practices are important predictors of predation by large carnivores. The elimination of the jaguar and puma was a common alternative (albeit, illegal) to resolve the conflict between large carnivores and human interests. The fact that an expansion of livestock breeding, and agriculture is underway to produce more food and wealth (SDG 2), while impeding the goal of biodiversity conservation (SDG15), should be an alarm call to reconcile both sustainable development objectives.

Most of the properties visited were small-scale livestock producers, meaning that losses due to predation or other causes have an important impact on production. Respondents reported an average 7% annual loss of livestock from 2010–2019 due to predation by jaguars and pumas. However, none of the respondents implemented bookkeeping practices to maintain a permanent record of livestock, which introduces some margin of uncertainty [21]. The lack of this information could result in overestimation of the damage caused by large carnivores [9]. In Brazil and Argentina, predation was found to be the main cause of livestock mortality, with annual losses of 0.02–2.83% reported in the Pantanal of Brazil [22], while Guerisoli et al. (2017) [23] reported annual losses of 3–9% in Argentinian Patagonia. In this study, cattle were the most abundant livestock and most affected by predation, similar to Guatemala [7]. Nonetheless, in one study area in Brazil, it was reported that abundance was not a determining factor for predation, since horses were the most predated by pumas, without being the most abundant. This indicates that selection may be influenced by the preference of the carnivore [8].

Our results indicate that diseases due to poor sanitary management and livestock theft are more critical challenges for livestock production than predation. Similarly, Castañeda (2009) [30] found that limited access to veterinary supplies, diseases, livestock theft, and floods are responsible for significant economic losses in the Muskitia Hondureña. Previous studies have also reported that malnutrition, disease, and poor management practices result in greater losses [39]. Inadequate sanitary management of herds and poor husbandry practices can facilitate exposure to diseases and large carnivore access to livestock [12,21].

Soto-Shoender and Giuliano (2011) [7] reported that livestock predation by large carnivores was more common at night. Similar results were obtained from our interviews, since most of the respondents assured that the jaguar and puma are dawn to nocturnal predators. However, Cavalcanti and Gese (2010) [12] reported that the jaguar does not select specific time periods to prey. Furthermore, it has been reported that livestock predation occurs both in times of drought [12] and during the rainy season [22]. Similarly, in our study, respondents reported livestock predation during both seasons. According to studies elsewhere, low availability of natural prey [11], and periods of livestock births [39] could be associated with increased predation.

Our results are consistent with previous studies indicating that livestock predation frequently occurs along forest edges, in grazing areas that are not closely monitored, and along riverbanks [8,39]. Predation events were also reported within forest cover and near human facilities and homes. Consequently, distance to the center of the community and distance to forest cover were important predictors in our selected model. Sites farther from human influence and closer to forest cover are more susceptible to predation by large carnivores. Soto-Shoender and Giuliano (2011) [7] reported that forest cover, distance to forest cover, bodies of water, and human settlements were important predictors of livestock predation in Guatemala, while in Brazil, predation occurrences have been more frequent in sites with high elevations and at greater distances from community centers [8]. The cases reported in different sites of Latin America show the need to understand that poverty, predator conservation, and adequate support for small-scale farmers are a triad that is not properly addressed in global policies.

In our study, the use of dogs on properties, bringing livestock closer to human facilities at night, and constant vigilance were some of the measures mentioned to mitigate predation. However, most of the grazing areas were adjacent to forest edges and there was little control over livestock due to the extensive management system (free grazing) used on the properties. This management system often results in the occurrence of wildlife on the property, including jaguars and pumas, or livestock in areas inhabited by wildlife, making it difficult to implement measures to mitigate livestock predation [21]. In order to implement effective measures to mitigate the conflict between livestock farming and top predator conservation, several actions can be taken, including night confinement of livestock during periods of vulnerability, use of guard dogs, use of visual and acoustic repellants, and electric fences [18,19,20,21]. Simply fencing livestock out and away from forest cover, and riparian brush and forest areas would reduce depredation and help maintain stream and river quality. It might require the use of either water tanks or constructed water retention ponds. Separating livestock and wildlife (including carnivores) through separate livestock-dedicated water sources has been recommended as a conflict-reducing tool [11,20].

There are examples of the efficacy and cost-effectiveness of predation deterrent practices. In ranches of 2401–22,000 ha with 400–18,000 head of cattle in the Paraguayan Chaco, Villalba et al. [20] and Polisar et al. [11] documented the cost-effectiveness of blinking systems of LED lights and varying combinations of electric fences, improved (tighter) livestock management, changes in spatial arrangement of livestock (maintaining a distance from forest areas), keeping cattle in more secure areas at night, and temporal concentration of birthing season [11,20,43]. Additionally, protecting natural prey from illegal hunting allows top predators to maintain healthy populations naturally. These measures were employed in pastures that had documented high rates of livestock predation by jaguars [43]. They not only stopped the losses in those areas, but they were also cost effective in four of six farms, with investment returns of 1.5, 2, 4, and 15 times the investment the control methods required [20]. These operations are larger scale with more resources than the farms in the Muskitia Hondureña, but several of these methods are replicable in Honduras. Working with livestock operations in the “Selva Lacandona” of Mexico that were all under 150 head [17], employed a similar evidence-based analysis of the efficacy of anti-predation techniques. The measures included training in large carnivore ecology, anti-depredation techniques, and animal husbandry, emphasizing night enclosures and electric fences in pastures that had suffered heavy losses. Across 11 ranches, the benefit–cost ratios ranged from 1.2 to 26.6, documenting efficacy in reducing losses, without lethal control of carnivores. While electric fences may or may not be difficult to sustain in the Muskitia Hondureña, night enclosures would not be. They proved effective in deterring predation in the buffer zone of the Maya Biosphere Reserve in Guatemala [44]. Relatively high densities of jaguars in proximity to human habitations, economic activities, and transportation routes are possible, but are best accomplished through attention to conflict and threat-reducing measures that harmonize economic and environmental priorities. High jaguar densities were found in a study area in Venezuela on a ranch managing about 10,000 head of cattle [45], with possible solutions, including forest blocks for natural prey (open area to forest ratio of 50:50), no hunting of natural prey, and tight livestock management (controlled reproduction, good nutritional status, and moving cattle among pastures and savannas on a seasonal basis and in response to conflicts). Although the management measures available to large ranches may be difficult to replicate in the small operations of the Muskitia Hondureña, in a section of the same bi-national JCU in the Muskitia Nicaragüense, improved livestock management, tighter herd control, better nutrition through silvopastoral systems and improved pastures, conservation agreements, and moderation of hunting, resulted in higher livestock productivity, the recovery of 800 km² of forests, increased bird diversity, no decreases in mammal diversity or abundance, and a drastic decline in human–jaguar conflict [46,47,48].

At present, non-lethal methods to reduce depredation are rarely practiced in the Muskitia Hondureña because most ranchers do not have knowledge of their feasibility and cost-effectiveness. In some cases, there is distrust of the efficiency of innovative methods or ranchers have already adopted a negative perception towards large carnivores [7,23]. In all these cases, well-designed pilot projects focused on chronic loss areas deploying the anti-predation techniques best adapted logistically and cost-wise to the area as well as documenting pre- and post-technique losses and cost–benefit ratios, may be able to overcome resistance. Despite the current tendency towards lethal control, many of the farmers we interviewed appreciate the natural world and would likely welcome pragmatic tools to improve coexistence.

Occasionally, opinion can be related to the age of the respondent [8,49] or schooling [10], but in our area the results indicated that the current opinions of how to reduce economic losses from predation were not dependent on the age or education of the respondent, nor the herd size on the property. Carnivore elimination was the most frequent method suggested by respondents, a solution obviously contrary to the goals of biological conservation and intact ecosystems in a biosphere reserve. Financial compensation for damage caused by large carnivores was another proposed alternative. However, that method has been widely criticized by conservationists since it can lead ranchers reducing their efforts to prevent predation, instead encouraging the occurrence of unverified losses and fraudulent claims [50], not to mention the difficulty of maintaining funding for such a mitigation effort in Honduras.

Although it has been reported that the elimination of large carnivores occurs in retaliation to livestock predation [12], our study found that the jaguar and puma are mainly eliminated because of the perceived risk of economic loss. The term ‘retaliatory killing’ has recently received overuse. Killing before experiencing losses to reduce risk likely occurs from the humid Muskitia of Honduras (Mesoamerica) to the semi-xeric Chaco in Paraguay (South America). It merits correspondingly proactive promotion of proven coexistence techniques that may diminish the pressure to kill.

Based on our results, aiming to reconcile SDG 2 and 15 should be a combined effort between the Departments of Agriculture and Environment. Until now, conservation strategies and food production initiatives are separated within ground-based government actions where poor farmers coexist with large predators in remote areas. We recommend that agents of governmental and non-governmental programs focus efforts on providing technical assistance to livestock ranchers in the Muskitia Hondureña, with the goals of (1) strengthening the sanitary status of their herds (incorporation of efficient livestock health programs) and ensuring their productivity; and (2) tightening up livestock management through better herd control, particularly at night, exclusion from forests, and protection of the most vulnerable classes of livestock [11,17,20,43]. Increased cattle production can contribute to supplying animal protein and milk to local communities, achieving SDG 2. Technical assistance should be prefaced by clear commitments from farmers that increased productivity will be limited in space, with zero spatial expansion, zero deforestation (in fact forest recovery can and should be a goal), with commitments to preserve natural habitats for carnivores and biodiversity, contributing to SDG 15. We recommend that the implementation of management systems that reduce the vulnerability of livestock be promoted. Cattle are most vulnerable to predation between birth and six months of age [21]. We recommend using confinement pens for young calves (at least the first three months of life) and their mothers. Given evidence from other studies [7,8,39] and that our results indicate that predation is more significant in proximity to forest cover and far from human settlements, if the pens are built close to the property’s house and have constant surveillance and/or dogs, the results should be more effective. This confinement system during periods of vulnerability could also help minimize livestock theft, which respondents indicated as the second greatest challenge of maintaining livestock.

Many livestock producers in Honduras and other Mesoamerican countries do not maintain records of their activities, either due to illiteracy or by choice. Ranchers should be encouraged to use livestock production records when possible, to have a written record of all sold, stolen, and dead livestock, both from natural and other causes [9]. Technical assistance can be accompanied by environmental education programs aimed at school children and ranchers, and involving ecology and biodiversity conservation, including the value and ecological role of top predators [8,51] found that education programs targeted at children also influenced their parents when they helped with the children’s homework. Multidisciplinary training is best, while a high-level of community participation and simple visual didactic material designed for people with low literacy [9] will increase accessibility and uptake. It is essential that these programs be long term to establish a baseline and then monitor and evaluate efficiency. Financial compensation is not recommended to mitigate economic losses from predation, since this measure has been unsustainable over time. An alternative to compensation programs is payment for environmental services [50], meaning payments for ensuring the conservation of the ecosystems occupied by the jaguar and puma. In this way, local interest in protecting these conservation icons is also encouraged. In some settings, rewards for camera trap photographs of large cats on farms have stimulated pride and changed attitudes [52]. In the Muskitia Hondureña, this could also be a possibility if local communities were encouraged to take part in conservation efforts and/or scientific research involving camera traps. Reconciling SDG 15 (biodiversity conservation and ecosystem protection) with farming activities that provide income and food, may be a challenge, but there are recorded experiences elsewhere showing that, with adequate commitments, it is possible.

## 5. Conclusions

The human–carnivore conflict has the potential to cause economic losses in livestock production. The average annual loss of 7% from 2010–2020 was attributed to predation by jaguars and pumas. However, diseases due to poor sanitary management and theft of livestock are more critical challenges. On the other hand, livestock predation is not random, but rather, related to landscape variables and human influence. Sites farther from human influence and closer to forest cover were more susceptible to predation. Nevertheless, studies across Latin America have shown that, through the implementation of appropriate management practices, it is possible to mitigate livestock predation and coexist with large carnivores. Finally, preventive killing of jaguars and pumas due to the perceived risk of economic loss was common in the study area. A change in the negative perception of ranchers towards large carnivores is essential to ensure their conservation. This study represents a baseline to understand the magnitude of the human–carnivore conflict with respect to livestock in one of the largest biosphere reserves in Mesoamerica. Achieving SDG 2 and 15 in the third decade of the XXI century must confront the fact that small-scale farmers are facing the same challenges that they have had for 200 years. Better governance and combined strategies for poverty alleviation, better livestock management, and proper care of protected areas and top predators are all mandatory priorities, not only in Honduras, but also in many remote areas of Latin America.

## Figures and Tables

**Table 1:** Description of the variables selected for analysis.

Variable	Range	Value	Analysis
Forest cover (%)	31–92	Continuous	GLM
Distance to forest cover (m)	0–237	Continuous	GLM
Distance to the center of the community (m)	532–20,094	Continuous	GLM
Property size (ha)	4–1410	Continuous	GLM
Herd size	6–153	Continuous	GLM
Herd size	6–50	Categorical	Fisher’s exact test
	51–100		
	>100		
Age class	23–34		
	35–49	Categorical	Fisher’s exact test
	50–64		
	>65		
Schooling	No schooling	Categorical	Fisher’s exact test
	Primary studies		
	Secondary studies		
	Superior studies		
Time of day	06:00–17:59	Categorical	χ^2^
	18:00–5:59		
Season	Drought	Categorical	χ^2^
	Rain		
Type of livestock	Cattle	Categorical	χ^2^
	Horses		
	Pigs		
	Sheep		

**Table 2:** Evidence evaluated by respondents to identify livestock predation by large carnivores and the sites where predation frequently occurred. Some respondents mentioned more than one category.

Categories	N	Percentage of Respondents
Evidence of predation		
Partially consumed body	9	13.24
Carnivore tracks	8	11.77
Claw marks	8	11.77
Drag marks	8	11.77
Neck and throat bites	8	11.77
Consumption of tongue and heart	7	10.29
Carnivore prowling	6	8.82
Hiding of prey	6	8.82
Skull fractures	4	5.88
Signs of struggle	3	4.40
Stress sounds	1	1.47
Total	68	100.00
Predation sites		
Forest edges	19	55.88
Grazing paddocks	7	20.59
Riverbanks	5	14.71
Forests	2	5.88
Near human facilities	1	2.94
Total	34	100.00

**Table 3:** Model selection statistics to test overall model fit.

Model Type	*K* ^1^	AICc ^2^	ΔAICc ^3^	*w* ^4^	Variables in Model
Landscape + human influence	3	55.97	0.00	0.29	Euclidian distance to forest cover + Euclidian distance to the center of the community
Landscape + human influence	4	56.44	0.47	0.23	Forest cover + Euclidian distance to forest cover + Euclidian distance to the center of the community
Landscape + human influence	3	57.21	1.24	0.16	Forest cover + Euclidian distance to the center of the community
Landscape + human influence + property profile	4	58.35	2.37	0.09	Euclidian distance to forest cover + Euclidian distance to the center of the community + herd size
Global Model (all parameters)	5	58.70	2.73	0.08	Forest cover + Euclidian distance to forest cover + Euclidian distance to the center of the community + herd size
Human influence + property profile	3	58.73	2.75	0.07	Euclidian distance to the center of the community + herd size
Landscape + human influence + property profile	4	59.64	3.66	0.05	Forest cover + Euclidian distance to the center of the community + herd size
Landscape	3	62.01	6.03	0.01	Forest cover + Euclidian distance to forest cover
Landscape + property profile	3	62.58	6.61	0.01	Euclidian distance to forest cover + herd size
Landscape + property profile	4	64.43	8.46	0.00	Forest cover + Euclidian distance to forest cover + herd size
Landscape + property profile	3	66.67	10.69	0.00	Forest cover + herd size

^1^*K* = number of parameters. ^2^ AICc = “corrected” Akaike’s information criterion for small samples. ^3^ ΔAICc = delta AICc differences. ^4^ *w* = Akaike weight.

## Data Availability

None of the data were deposited in an official repository. The access rights to data, software or model are available to reviewers.

## Supplementary Materials

The following supporting information can be downloaded at: https://www.mdpi.com/article/10.3390/ani12040479/s1, Depredación de ganado por jaguar (*Panthera onca*) y puma (*Puma concolor*): Un estudio de caso en La Mosquitia Hondureña.
