# Supplementary material for: Livestock–Carnivore Coexistence: Moving beyond Preventive Killing
Source: Animals (Basel). 2022 Feb 15;12(4):479. doi: 10.3390/ani12040479 (PMC8868381; doi:10.3390/ani12040479)
Supplement: Supplementary file 1 [file animals-12-00479-s001.zip › animals-1543401-supplementary.pdf]

Depredación de ganado por jaguar (*Panthera onca*) y puma (*Puma concolor*): Un estudio de caso en La Mosquitia Hondureña

Nombre de la comunidad:

____

Perfil del entrevistado

1. Rol en la propiedad

☐ Propietario ☐ Empleado ☐ Otros_____________

2. Tiempo de residencia en la propiedad

☐ >20 años ☐ 10-20 años ☐ 5-10 años ☐ <5 años

3. Edad _____________

4. Sexo

☐ Femenino ☐ Masculino

5. Escolaridad

☐ Escuela primaria ☐ Secundaria ☐ Superior ☐ No estudios

6. Grupo indígena en el cual se autoclasifica

☐ Misquito ☐ Tawahka ☐ Pech ☐ Garífuna ☐ Otros _____________

Perfil de la propiedad

7. Coordenadas UTM:_____________ _____________

8. Tamaño de la propiedad (manzanas):_____________

9. Uso de la propiedad:

☐ Ganadería ☐ Cultivos ☐ Forestal ☐ Recursos minerales ☐ Bosque (sin uso)

☐ Otros ____________

Describa el uso de la propiedad en proporción (tamaño o %):

____________Ganadería
____________Cultivos
____________Forestal
____________Recursos minerales
____________Bosque (sin uso) =100%

Desafíos de la producción ganadera

10. Tipos de animales domésticos en la propiedad:

☐ Bovino (leche, carne, doble propósito) ☐ Equino ☐ Porcino ☐ Caprino ☐ Ovino ☐ Avícola ☐ Perros ☐ Otros_________

11. Proporcione un estimado de cuántos animales domésticos hay en esta propiedad y las pérdidas que experimenta por año. Si los números varían cada año, describa los números mínimos y máximos estimados por año.

Tipo de ganado	Cantidad de ganado por año		Pérdidas de ganado por año (todas las causas)	
	Min	Max	Min	Max
Bovino				
Equino				
Porcino				
Caprino				
Ovino				
Avícola				
Perros				
Otros				

12. Indique los desafíos principales de su producción ganadera e indique cuál de ellos es el más importante.

_____________Depredación
_____________Enfermedad
_____________Inundación
_____________Preparto
_____________Postparto
_____________Robo
_____________Sequía
_____________Serpiente
_____________Otros

13. ¿Ha observado depredación de ganado en el pasado?

☐ Si ☐ No

14. Tipo de manejo en su producción ganadera

☐ Extensivo ☐ Confinamiento por la noche ☐ Confinamiento por época

☐ Confinamiento total ☐ Otros_____________

*****Si el entrevistado proporciona datos de depredación se continuará con la siguiente parte de la encuesta/entrevista.**

Depredación de ganado por grandes carnívoros

15. ¿Considera usted que la principal causa de pérdida de ganado en su producción es por la depredación?

☐ Si ☐ No

16. De los animales domésticos reportados en la tabla anterior, proporcione un estimado de cuántas unidades ganaderas han sido depredadas por año y el tipo de carnívoro que considera responsable de los eventos. Si los números varían cada año, describa los números mínimos y máximos estimados por año.

Tipo de ganado	Unidades ganaderas depredadas por grandes carnívoros		Tipo de carnívoro ______		Tipo de carnívoro ______	
	Min	Max	Min	Max	Min	Max
Bovino						
Equino						
Porcino						
Caprino						
Ovino						
Avícola						
Perros						
Otros						

Año con mayores eventos de depredación: ______________

17. ¿Usted considera que la depredación de ganado ha aumentado o disminuido en el tiempo?

☐ Aumentado ☐ Disminuido

18. ¿Qué factores toma en cuenta para concluir que su ganado fue depredado por grandes carnívoros?

____

____

19. ¿Como evalúa si su ganado murió por causa natural y luego fue consumido por grandes carnívoros o efectivamente la depredación causó la muerte del animal?

____

____

20. ¿Puede usted diferenciar entre ataque de jaguar y puma?

☐ Si ☐ No

21. De ser sí, ¿qué factores toma en cuenta?

____

____

22. Época del año en que ha observado más eventos de depredación a su ganado

☐ Sequías ☐ Lluvias

23. Tiempo de día/noche en que ha observado más eventos de depredación a su ganado

____

24. Localidades de su propiedad en donde ha observado más eventos de depredación a su ganado

____

____

Percepciones y metodología letal/no letal para mitigar la depredación de ganado

25. ¿Que prácticas implementa para mitigar la depredación de ganado?

____

____

26. ¿Ha observado alguna reducción de la depredación luego de implementar estas prácticas?

☐ Si ☐ No

27. ¿Cómo cree usted que se podrían reducir las pérdidas económicas por depredación de ganado en su propiedad?

☐ Mejorar prácticas de manejo ☐ Compensación financiera ☐ Eliminar al carnívoro
☐ Otros____________

28. ¿Sabe de alguien que haya eliminado a algún carnívoro?

☐ Si ☐ No

29. De ser sí, ¿cuál cree usted que es la razón para eliminar a un carnívoro?

☐ En respuesta a depredación de ganado en el pasado ☐ Para prevenir depredación de ganado en el futuro ☐ Para proteger a las personas de ataques

☐ Por dinero ☐ Otros____________

30. De ser sí, ¿qué tipo de carnívoro eliminaron?

☐ Jaguar ☐ Puma ☐ Jaguar y pum
